# Modulation of Digestive Enzyme Activities and Intestinal γ-Proteobacteria in Gilthead Sea Bream Fed High-Fat Diets Supplemented with HIDROX^®^ Olive Oil Extract

**DOI:** 10.3390/ani15142102

**Published:** 2025-07-16

**Authors:** Irene García-Meilán, Sara Balbuena-Pecino, Manel Montblanch, Sara Ramos-Romero, Ramón Fontanillas, Joaquim Gutiérrez, Encarnación Capilla, Isabel Navarro, Ángeles Gallardo

**Affiliations:** 1 Departament de Biologia Cel·lular, Fisiologia i Immunologia, Facultat de Biologia, Universitat de Barcelona, Av. Diagonal 643, 08028 Barcelona, Spain; sarabalpe14@gmail.com (S.B.-P.); mmontblanch@ub.edu (M.M.); sara.ramosromero@ub.edu (S.R.-R.); jgutierrez@ub.edu (J.G.); ecapilla@ub.edu (E.C.); mnavarro@ub.edu (I.N.); mgallardo@ub.edu (Á.G.); 2 Nutrition & Food Safety Research Institute (INSA-UB), Maria de Maeztu Unit of Excellence, 08921 Santa Coloma de Gramenet, Spain; 3 Skretting Aquaculture Research Centre (ARC), Sjøhagen 3, 4016 Stavanger, Norway; ramon.fontanillas@skretting.com

**Keywords:** lipase, α-amylase, protease activity, zymography, trypsin, chymotrypsin, microbiota

## Abstract

High-fat diets are commonly used in fish farming due to their protein-sparing effect, contributing to reduced production costs. However, this practice may have adverse effects such as metabolic impairment and inflammation. These problems can be assessed in two ways: by developing functional diets or using food restriction, which leads to compensatory growth. The present study characterized digestion in gilthead sea bream fed a high-fat diet in the presence (HT) or absence (HF) of an olive oil polyphenol as an additive, hydroxytyrosol, under two different dietary regimes: feeding to satiation (ST) or at a 40% restriction (R). Digestive enzyme activities, specifically trypsin-like activities, were mainly upregulated by dietary treatment (HT). In contrast, restriction effects mainly appeared during digestion in the pyloric caeca, where a significant rise in chymotrypsin-like activities was detected. Moreover, those fish tended to have an increased relative intestinal length compared to those fish fed at a standard ration. Feed restriction enhanced the growth of γ-Proteobacteria in pyloric caeca and proximal intestinal regions, without altering their population in the distal intestine. Overall, it is suggested that hydroxytyrosol inclusion at a standard ration could improve digestion processes in gilthead sea bream fed high-fat diets under healthier conditions than without this additive.

## Simple Summary

The need to reduce production costs in aquaculture has led to the use of aquafeeds with high lipid content, which can negatively impact fish welfare. The present study aims to address these problems by, on one hand, using a feed additive of olive oil origin, and, on the other hand, reducing the given ration to assess digestive functions and microbiota modulation. Overall, digestive activities were upregulated by the inclusion of the additive. In addition, dietary restriction directly affected intestinal length, resulting in longer intestines in those fish fed a restricted diet compared to those fed a standard ration. Moreover, restriction enhanced the presence of Proteobacteria, the most common gut microorganism found in marine fish species, in the anterior intestinal region. These findings suggest that fish fed high-lipid diets containing HIDROX^®^, a hydroxytyrosol-rich additive, and fed at a standard ration show improved digestion and overall health.

## 1. Introduction

Nowadays, there are different challenges that aquaculture must face, and one of the most important is producing a cheap and affordable protein source to feed the increasing human population [1]. To fulfill this demand, the supply of aquafeed ingredients is expected to reach 30 million tonnes by 2030 [2]. This framework has changed aquafeed production trends over the past decades, from the use of traditional ingredients, including fish meal and fish oil [1,3], to the incorporation of agriculture-based ingredients and/or fish offal and trimmings, among other by-products. These are cheaper than traditional aquafeed sources [4] due to their independence from wild fish stocks and limited supplies. Despite this, the use of plant-based ingredients or aquaculture by-products comes with their own set of problems, such as the presence of anti-nutritional factors, and even deficient amino and/or fatty acid profiles that may lead to reduced growth, intestinal inflammation, microbiota impairment, and immunodeficiency favoring disease outbreaks [5,6,7,8,9]. As fish can metabolize lipids as an energy source rather than carbohydrates [10], the use of high-fat diets (HFDs) in fish feeding has increased in recent years, contributing to enhanced fish growth and reduced production costs due to the protein-sparing effect of lipid intake [11,12,13]. However, the administration of HFD could also have negative effects due to metabolic impairment, leading to increased fat deposition in the liver, reduced lipid catabolism, and inflammation [10]. Another strategy to consider, related to food management, is food restriction, which could improve fish performance through compensatory growth [14,15]. Food restriction is a natural process that takes place during an animal’s lifespan, triggering metabolic adaptations while improving feeding efficiency by optimizing the digestive process, but it also has physiological consequences in organisms, particularly through the generation of oxidative stress [16]. Within this framework, sustainable aquaculture development needs to be improved by formulating functional diets that include natural products or enhanced plant extracts, which have positive effects on the productivity and health of farmed species [17,18,19,20,21,22,23]. But the main drawback is the variability of both the bioactive compound composition and their effects *in vivo* [24,25].

In the Mediterranean countries, olive (*Olea europaea*) oil and its by-products (olive mill water, olive pomace, waste olive cake, and olive leaf) are among the richest sources of polyphenols [26]. In addition to being high in monounsaturated fatty acids, they form a complex matrix containing hydroxytyrosol, tyrosol, their derivatives, and oleuropein [27]. Thus, olive oil and its by-products have shown intestinal anti-inflammatory activity in humans [28,29], which is associated with hydroxytyrosol and tyrosol. These metabolites can also enhance the growth of gut microbiota, such as *Lactobacillus acidophilus* [30,31]. Similar results were also found in mouse studies, where microbial diversity was increased after olive oil dietary inclusion [32,33]. Moreover, in humans and rats, undigested polyphenols reaching the colon are metabolized by gut microbiota into absorbable compounds (i.e., phenylacetic and phenylpropionic acid derivatives, protocatechuic and hydroxybenzoic acids) that provide energy and nutrients to the body [34,35]. In fish, olive oil or its by-products have antioxidant and antimicrobial properties that contribute to improving the immune status or act as immunostimulants, boosting fish growth and feed efficiency [36,37,38,39]. However, depending on their concentrations, polyphenols can exhibit prooxidant properties [40], which can negatively impact fish performance [41,42]. So, the effect on fish largely depends on the type of added polyphenol and the level of inclusion [43]. The incorporation of olive juice extract into aquafeeds with moderate lipid content (18%) has been found to enhance immune properties, gut health and functionality, and somatic growth in gilthead sea bream (*Sparus aurata*) and black sea bream (*Acanthopagrus Schlegelii)* [44,45]. In addition, the anti-obesogenic properties of such polyphenols (i.e., hydroxytyrosol) were also demonstrated in rainbow trout (*Onchorhynchus mykiss*) and zebrafish (*Danio rerio*) [46]. Despite this, little is known about their effects at the intestinal level in fish [47,48].

The gut microbiota is a complex community of microorganisms residing in the intestinal tract of fish, where it plays a crucial role in nutrient processing, immune modulation, and overall health maintenance [49]. In marine fish, Proteobacteria are among the most abundant symbionts, comprising approximately 80% of their gut microbiota, largely due to their highly flexible metabolic properties [50,51]. This phylum, together with Firmicutes and Actinobacteria, is the most abundant bacterial phylum in the gilthead sea bream intestine [52,53]. Several factors, such as the host genetics, developmental stage, water quality, environmental conditions, and feed, can influence gut microbiota [54,55]. In this sense, Nikouli et al. [56] found that the dietary administration of different lipid sources did not significantly affect the composition of gut bacteria in the early life stages of Atlantic salmon (*Salmo salar*). However, high-fat diets have been shown to alter the proportions of several bacterial groups in the gut microbiota of various fish species, like hybrid yellow catfish (*Tachysurus Fulvidraco ♀ × Pseudobagrus Vachellii ♂*) [57] and Nile tilapia (*Oreochromis niloticus*) [58]. Microbiota imbalance, also known as dysbiosis, has been related to diverse metabolic changes in the host, including weight gain and fat accumulation, increased risk of infection, and inflammation [59]. Overall, these studies highlight the impact of HFD on the gut microbiota of fish, and perhaps the addition of dietary supplements, such as a hydroxytyrosol-rich olive oil extract, like HIDROX^®^, may help mitigate any negative effects and promote a healthy gut microbiota.

This study is part of a broader experiment, the results of which concerning growth and lipid metabolism have been recently published [60,61]. These results suggested that gilthead sea bream fed an HFD containing a hydroxytyrosol-rich extract from olive juice for 8 weeks had a higher growth potential due to an increase in the plasma insulin-like growth factor 1 (IGF-1) and upregulated gene expression of markers related to musculoskeletal growth compared to those fish fed an HFD without the additive. In addition, some of these hydroxytyrosol effects were maintained even in fish fed under 40% of dietary restriction [61]. Moreover, it was demonstrated that gilthead sea bream fed with these diets had reduced free fatty acid plasma levels and liver lipid metabolism, thus preventing diet-induced steatosis, and increased muscle lipid content and peroxidation [60]. However, regarding growth performance, only differences due to feed restriction were found between groups, regardless of the given diet, showing a reduction of 11.42% in body weight compared to those fed the standard ration [61]. Nevertheless, there is no available information about the effects of incorporating this additive on digestion. This study aimed to evaluate the digestive process in gilthead sea bream (*Sparus aurata*) fed an HFD, administered at two rations: standard (morning and afternoon feeding) and restricted (morning feeding only). The goal was to assess the impact of an olive juice extract rich in hydroxytyrosol as a dietary additive evaluated at 24 and 5 h post-feeding and to provide an initial characterization of the gut microbiota under these feeding conditions.

## 2. Materials and Methods

### 2.1. Fish and Experimental Design

Two hundred forty gilthead sea bream (*Sparus aurata*) juveniles (50.11 ± 8.12 g) from Piscimar (Burriana, Spain) were acclimated to the facilities of the Faculty of Biology at the University of Barcelona (Spain) for one month. Fish were fed ad libitum twice daily with a commercial diet (18% lipids, 48.5% protein and 18.5 MJ/kg digestible energy) (Optibream Skretting, Burgos, Spain) and kept at 23 ± 1 °C, pH 7.9 and 12L/12D photoperiod in a semi-closed recirculation system with physical and biological filters, ozone, and continuous aeration with a 35‰ weekly sea water renewal rate. After that period, fish with an initial body weight of 80.8 ± 1.4 g were randomly distributed in twelve tanks (4 of 400 L with 30 fish per tank and 8 of 200 L with 15 fish per tank, due to the particular tank distribution at the UB fish facility), all of them with the same biomass density (6.06 kg/m^3^). An experimental high-fat diet (HF), with 24% dry matter of lipid origin (1:1 rapeseed oil: fish oil), 46.8% protein, and 23 MJ/kg of digestible energy, was formulated and produced by Skretting ARC (Stavanger, Norway) (Table 1).

Fish were fed this diet one week before starting the trial to habituate the fish, identify visual acceptance, and establish the standard ration (ST) that would correspond to satiety in the present experiment. In addition, the diet was also formulated including an additive (HT) with 1.66 g HIDROX/kg feed (0.52 g HT/kg feed), using specifically a commercial olive oil extract rich in hydroxytirosol (HIDROX^®^ certificate of analysis 12-190403-000: >12% simple and total polyphenols: 3.136% hydroxytyrosol, 0.216% oleuropein, and 0.408% tyrosol, provided by Oliphenol LLC. (Hayward, CA, USA)). Regarding nutrient composition, both diets were the same, except for starch content, since wheat was reduced due to additive inclusion (Table 1).

**Figure 1:**
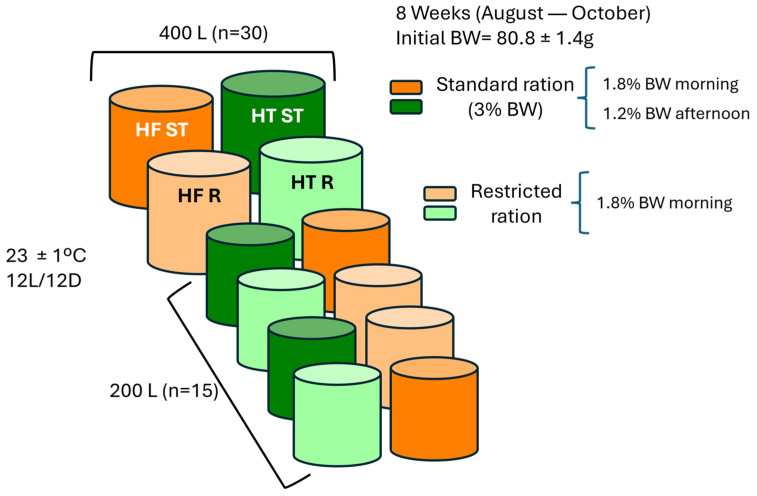
Experimental design in the Faculty of Biology facilities. HF ST: gilthead sea bream fed high-fat diet at standard ration; HT ST: gilthead sea bream fed high-fat diet supplemented with hydroxityrosol at standard ration; HF R: gilthead sea bream fed high-fat diet at restricted ration; HT R: gilthead sea bream fed high-fat diet supplemented with hydroxityrosol at restricted ration; BW: body weight.

The experimental trial was conducted in triplicate tanks for 8 weeks (from the end of August to the end of October), and dietary treatments were administered at two different rations: standard (3% of body weight (BW)) and restricted (1.8% of BW, 40% of restriction). Thus, four experimental groups were established: two with standard feeding (HF ST and HT ST) and two groups under restriction (HF R and HT R) (Figure 1). All groups received the same ration in the morning meal (1.8% BW), whereas in the afternoon, only those in the standard ration were fed (1.2% BW). The daily ration was adjusted every two weeks according to the weight gain of the fish.

The Ethics and Animal Care Committee of the University of Barcelona (permit numbers CEEA 34/20 and DAAM 11251) approved all animal handling procedures, following the Spanish and Catalan governments’ principles and legislation, and complied with the Guidelines of the European Union Council directive.

### 2.2. Sampling Procedures and Preparation

At the end of the growth period, two samplings were performed at 24 and 5 h post-feeding after the morning meal. Ten fish per treatment were deeply anesthetized (MS-222, Sigma, Madrid, Spain), weighted, measured, and sacrificed by sectioning the spinal cord. Moreover, the intestinal length of each fish, excluding pyloric caeca, was measured to calculate relative intestinal length (RIL) according to the formula mm/g fish.

Pyloric caeca and proximal intestine samples were collected, rapidly frozen in liquid nitrogen, and maintained at −80 °C until analysis. Samples from each intestinal section were thawed on ice, and the intestinal duct pH was measured (Crison Micro pH 2000, Barcelona, Spain). After that, individual samples were homogenized in Tris-HCl solution (50 mM, pH 7.5) using a Precellys Evolution^®^ Homogenizer (6500 rpm; 3 × 20 s with two breaks of 20 s; 4 °C) combined with Cryolys^®^ as a cooling system (Bertin Technologies, Montigny-le-Bretonneux, France). After a 15 min centrifugation (2400 rpm; 4 °C; Eppendorf 5418R, Hamburg, Germany), supernatants were stored at −80 °C until digestive enzyme activities and zymography analyses were performed. To obtain microbiota samples, fish were starved for 24 h before sampling. Individual samples of pyloric caeca and proximal and distal intestines were collected under sterile conditions and kept in cryovials at −80 °C until DNA extraction and microbiota analysis.

### 2.3. Digestive Enzyme Analysis

A kinetic assay was conducted to measure α-amylase and lipase activities in both intestinal segments according to the manufacturer’s instructions (ref. 41201, and 1001275, Spinreact, Sant Esteve d’en Bas, Girona, Spain) at 25 ± 0.5 °C using a microplate scanning spectrophotometer (Tecan Infinite 200 PRO, Tecan, Grödig, Austria). Briefly, for α-amylase, the rate of 2-chloro-4-nitrophenol formation at 405 nm was measured. For lipase, the rate of methylresofurin formation, measured at 580 nm, was proportional to the catalytic concentration of lipase in the sample. For both enzymes, one international unit (IU) is defined as the enzyme that hydrolyzes 1 μmol of substrate per min under standard conditions, and enzyme activities are expressed as mU per mg of protein.

Total protease activity (TPA) was measured using the casein-hydrolysis method described by Moyano and modified by Santigosa et al. [62]. Bovine trypsin (Sigma Aldrich, Madrid, Spain, T9935, 12100 BAEE U·mg protein^−1^) was used as a standard. Briefly, the samples and standards were reacted for 30 min with 1% casein buffer (50 mM Tris-HCl) at the pH of the intestinal duct, and the reaction was stopped by adding 12% trichloroacetic acid. Blanks were established for each sample and standard. After that, the reacted samples were centrifuged (5 min, 7500 g, 4 °C), and supernatant absorbance was determined at 280 nm (Tecan Infinite 200 PRO, Tecan, Grödig, Austria). TPA was calculated as BAEE units per mg of protein.

Protein quantification in sample homogenates from both intestinal segments was performed using the Bradford method (1976), using bovine serum albumin as a standard.

Individual trypsin-like and chymotrypsin-like activities were characterized by zymography according to the method described by García-Carreño et al. [63], modified by Santigosa et al. [62]. Homogenates were combined (4:1, *v*/*v*) with water or different inhibitor solutions selected according to Moyano et al. [64]: Nα-p-tosyl-L-lysin chloro-methyl ketone (TLCK) as a trypsin-like activity inhibitor and N-tosyl-L-phenyl-chloromethyl ketone (TPCK) and N-CBZ-L-phenyl-chloromethyl ketone (ZPCK) as chymotrypsin-like activity inhibitors, and they were allowed to react for 45 min at room temperature (RT). After, samples plus loading buffer (*v*/*v*) and a commercial molecular weight marker (Amersham GE Healthcare, Amersham, UK, RPN800E, 12,000–225,000 Da) were loaded on a 12.5% SDS-PAGE gel. The electrophoresis was performed on a Mini-PROTEAN^®^ Tetra Cell power supply at a constant current of 15 mA per gel for ~120 min at 4 °C. Once finished, the gels were incubated under agitation with 50 mM Tris-HCl buffer containing 2% casein at the pH of the intestinal duct for 30 min at 4 °C, and thereafter for 90 min at RT. A methanol–acetic acid–water (40:10:40) solution with 0.1% Brilliant Blue Coomassie R-250 was used to stain the gel for 25 min, followed by a 10 min destaining step with the same solution without colorant; both procedures took place under agitation.

Once proteolytic characterization was complete, zymogram images were processed by the GelDoc GO Imaging System (Bio-Rad Laboratories, Inc., Hercules, CA, USA), followed by an analysis with Image Lab software (Version 6.1.0 build 7 Standard Edition. © 2020, Bio-Rad Laboratories, Inc., Hercules, CA, USA). Each protease activity was quantified individually; however, in the results section, protease activity will be shown in two groups: trypsin-like and chymotrypsin-like activities.

### 2.4. Microbiota from Gut Mucosa

At the end of the study and 24 h post-feeding, the proportions of bacterial subgroups in the gut mucosa were estimated from basal DNA by quantitative real-time PCR (qPCR). DNA was extracted from the intestinal mucosa according to Castro et al. [65], and its concentration was quantified using a Nanodrop 8000 Spectrophotometer (Thermo Scientific, Waltham, MA, USA). qPCR was carried out in triplicate on diluted DNA samples (20 ng/µL) and the reactions were paralleled by analysis of a non-template control (water) and a positive control (*E. coli* M15 for Proteobacteria and Enterobacteriaceae, *Micrococcus luteus* for Actinobacteria, *Ruminococcus productus* for Firmicutes). The qPCR experiments were conducted in Hard-Shell^®^ 384-Well PCR Plates (HSP3801, Bio-Rad Laboratories, Hercules, CA, USA) on the CFX384^TM^ Real-Time System (Bio-Rad Laboratories, Hercules, CA, USA). The qPCR cycling conditions were 10 s at 95 °C, then 45 cycles of 5 s at 95 °C, 30 s at primer-specific annealing temperature (AN; Table 2), and 30 s at 72 °C (extension).

Following amplification, to determine the specificity of the qPCR, melting curve analysis was carried out by treatment for 2 s at 95 °C and 15 s at 65 °C, followed by a temperature gradient up to 95 °C at 0.11 °C/s, with five fluorescence recordings per degree Celsius. The relative DNA abundances for the different bacteria genes were calculated from the second derivative maximum of their respective amplification curves (Cp), according to the equation [DNAa]/[DNAb] = 2Cpb − Cpa [69]. Total bacteria were normalized as 16S rRNA gene copies per mg of wet mucosa samples (copies per mg).

### 2.5. Statistical Analysis

Shapiro–Wilk and Levene’s test were applied to test data normality and homoscedasticity. Digestive enzyme activity data did not achieve normality, and Kruskal–Wallis H followed by all pairwise comparisons using the U-Mann–Whitney non-parametric tests were applied to detect significant differences between experimental groups using the statistical software SPSS Statistics v25.0 (SPSS Inc., Chicago, IL, USA). Total bacteria between intestinal segments were evaluated by Kruskal–Wallis and Dunnett’s tests. The intestinal bacterial subgroups were evaluated by two-way ANOVA and Sidak’s multiple comparisons tests for mean comparison (SPSS Statistics v25.0). Differences were considered significant when *p* < 0.05. GraphPad 7.0 (GraphPad Software Inc., San Diego, CA, USA) was used for the graphics.

## 3. Results

### 3.1. Digestive Enzyme Activities and Relative Intestinal Length

**Figure 2:**
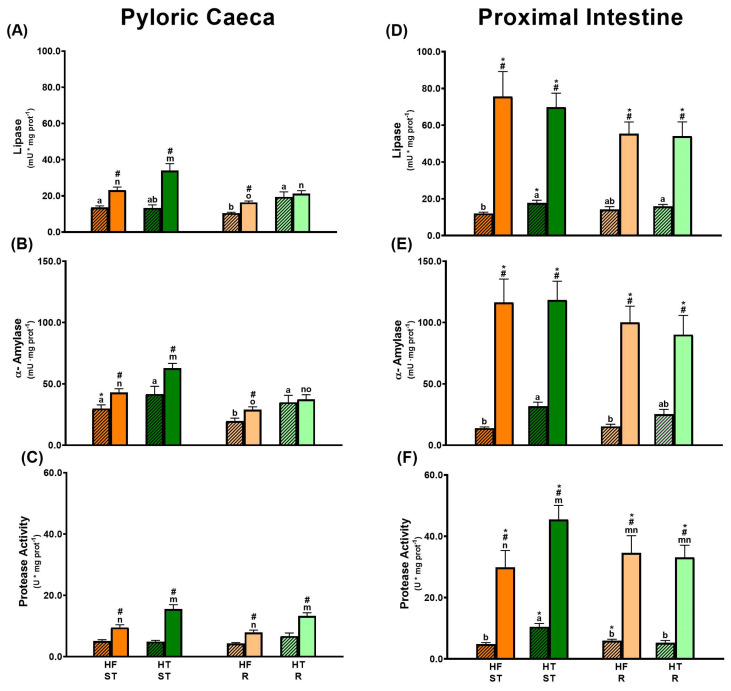
Lipase, α-amylase, and total protease enzyme activities in pyloric caeca ((**A**), (**B**), and (**C**), respectively) and proximal intestine ((**D**), (**E**), and (**F**), respectively) of gilthead sea bream fed the experimental diets for 8 weeks, sampled at 24 h (patterned bars) and 5 h post-feeding (smooth bars). Experimental conditions have the same color code as in Figure 1. Data are shown as mean ± S.E.M. (n = 10). Comparisons between experimental groups were performed using the Kruskal–Wallis test followed by the U-Mann–Whitney non-parametric test (*p*-value < 0.05). Letters show significant differences between experimental groups: from a to b in pyloric caeca, and from m to o in proximal intestine, where # indicates a significant difference between post-feeding times. Moreover, asterisks show significant differences between intestinal regions at the same postprandial time.

**Figure 3:**
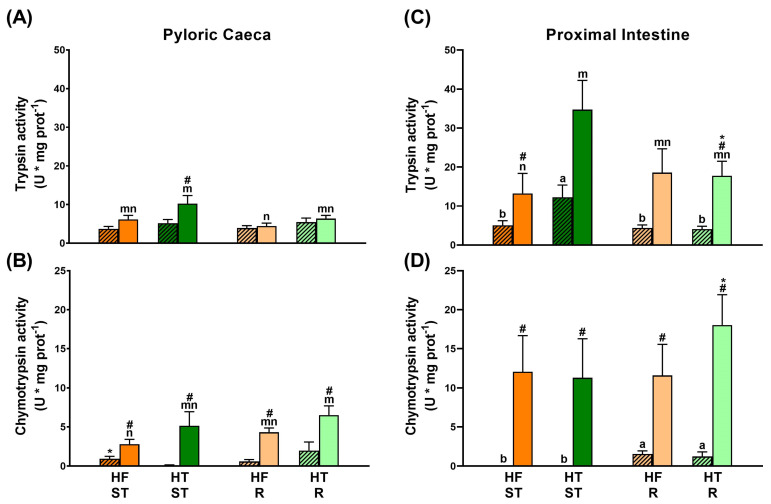
Trypsin-like and chymotrypsin-like activity in pyloric caeca (**A**) and (**B**), respectively) and proximal intestine (**C**) and (**D**), respectively) of gilthead sea bream fed the experimental diets for 8 weeks, sampled at 24 h (patterned bars) and 5 h post-feeding (smooth bars). Experimental conditions have the same color code as in Figure 1. Data are shown as mean ± S.E.M. (n = 10). Comparisons between experimental groups were performed using the Kruskal–Wallis test followed by the U-Mann–Whitney non-parametric test (*p*-value < 0.05). Letters show significant differences between experimental groups: from a to b in pyloric caeca, and from m to n in proximal intestine, where # indicates significant differences between post-feeding times. Moreover, asterisks show significant differences between intestinal regions at the same postprandial time.

Results regarding digestive activity (Figure 2 and Figure 3) are presented by comparing gilthead sea bream fed the HF ST diet (orange bars), used as a reference group, with the other experimental conditions: HT ST (green bars), HF R (light orange bars), and HT R (light green bars). Moreover, bars with a pattern represent anticipatory activities (24 h post-feeding), whereas smooth bars represent activities 5 h post-feeding.

Gilthead sea bream showed significantly higher lipase, *α*-amylase, and protease activity 5 h after the morning feeding compared to just before the meal (*p*-value ˂ 0.05) (Figure 2). Additionally, at 5 h post-ingestion, a marked regionalization was observed, with significantly greater digestive enzyme activities in the proximal intestine than in the pyloric caeca. Thus, at that time post-ingestion, digestion is occurring mainly in the proximal area of the intestine.

Zymography detected three bands with trypsin-like activity (T: 90, 60, and 55 kDa) and six with chymotrypsin-like activity (C: 50, 30, 25, 21, 17, and 15 kDa) (Table 3). The tables also show that just before feeding, in both the pyloric caeca and proximal intestine, the percentage of trypsin-like activity was much higher than that of chymotrypsin-like, with trypsin/chymotrypsin ratios not lower than 2.85. In contrast, the trypsin/chymotrypsin ratios at 5 h post-ingestion were lower, ranging from 0.89 and 2.51.

#### 3.1.1. Effects of Feeding Standard Ration (Ad Libitum) with HF and HT (HF ST Versus HT ST)

In HT ST gilthead sea bream compared with HF ST, an increase in lipase and α-amylase activities was detected both in pyloric caeca during digestion (*p*-value = 0.038 and *p*-value ˂ 0.001, respectively) and in the proximal intestine before feeding (*p*-value = 0.002 and *p*-value ˂ 0.001, respectively) (Figure 2A,B,D,E). Moreover, HT ST gilthead sea bream showed a significant upregulation in TPA after feeding in the pyloric caeca (*p*-value ˂ 0.001), and both before and after feeding in the proximal intestine (*p*-value ˂ 0.001 and *p*-value = 0.023, respectively) (Figure 2C,F). In this late intestinal segment, the increases in TPA were related to greater trypsin-like activity (*p*-value = 0.021), without significant regulation of chymotrypsin-like activity (Figure 3).

Table 3 shows the percentages of TPA for each of the individual protein proteases described. Feeding to satiety with the HT-supplemented diet upregulated the 60 kDa trypsin-like activity (*p*-value = 0.021) and downregulated the 50 and 30 kDa chymotrypsin-like activities in the pyloric caeca just before the morning intake (*p*-values 0.004 and 0.040, respectively), although the overall TPA of these animals was not modified.

On the other hand, at 5 h postprandial in the proximal intestine, the 30 and 25 kDa chymotrypsin-like activities decreased in percentage (*p*-values 0.036 and 0.021, respectively), modifying the trypsin/chymotrypsin ratio in both cases. In contrast, this ratio did not change in the pyloric caeca at 5 h post-ingestion, nor in the proximal intestine just before morning feeding.

These changes in digestive enzyme activities in gilthead sea bream fed to satiety with the HT diet can be related to the optimization of dietary digestion when hydroxytyrosol-rich extract is included in the diet. Finally, gilthead sea bream fed to satiety with the HF and HT diets showed similar RILs (0.59 ± 0.03 and 0.59 ± 0.04, respectively).

#### 3.1.2. Effect of Restriction When Feeding with HF (HF ST Versus HF R)

Restricted feeding with the HF diet, supplying the same morning ration to the fish and depriving them of the afternoon ration, caused a downregulation of anticipatory and postprandial lipase and α-amylase activities in the pyloric caeca (*p*-values = 0.003, 0.008, 0.002 and 0.001, respectively) while maintaining protease activity in this intestinal segment. In contrast, digestive activity was not modulated by restriction for any of the three digestive enzymes studied in the proximal intestine (Figure 2).

Although total protease activities were similar in animals on restricted feeding versus those fed the HF diet to satiation, individual protease activities were modified by the effect of food restriction (Table 3). Thus, the percentage of the 60 kDa band decreased in the proximal intestine just before feeding (*p*-value < 0.001), and the activities of the 55, 50, 30, and 25 kDa bands increased (*p*-value < 0.001, *p*-value < 0.001, *p*-value = 0.023, and *p*-value = 0.23, respectively), inducing a decrease in trypsin-like activity relative to chymotrypsin-like activity.

On the other hand, at 5 h post-feeding in the pyloric caeca, a reduction in trypsin-like activity (90, 60, and 55 kDa bands, *p*-values = 0.002, 0.015, and 0.001, respectively) and an increase in chymotrypsin-like activity (50, 30, 25, and 21 kDa bands, (*p*-values < 0.001, <0.001, <0.001, and 0.011, respectively) were observed.

In addition, HF R gilthead sea bream tended to increase their RIL compared with HF ST-fed fish (0.63 ± 0.04 versus 0.59 ± 0.03, respectively). Therefore, considering the significantly lower growth of these fish, their attempt to increase RIL, the maintenance of protease activity, and the decline in the trypsin/chymotrypsin ratio, the data showed that food restriction in this group is excessive.

**Table 3:** Total protease activity (TPA), individual trypsin-like (grey rows) and chymotrypsin-like (white rows) activities, and total trypsin-like and chymotrypsin-like activities in pyloric caeca (A) and proximal intestine (B), characterized by zymography in gilthead sea bream fed the experimental diets for 8 weeks, sampled at 24 h and 5 h post-feeding. Data are shown as mean ± S.E.M. (n = 10). Comparisons between experimental groups were performed using the Kruskal–Wallis test followed by the U-Mann–Whitney non-parametric test (*p*-value < 0.05). Letters showed significant differences between experimental groups: from a to e in pyloric caeca and from m to q in proximal intestine. Asterisks show significant differences between intestinal regions at the same postprandial time (*p*-value < 0.05).

(A) Pyloric Caeca	HF ST		HT ST		HF R		HT R	
Post-Feeding	24 h	5 h	24 h	5 h	24 h	5 h	24 h	5 h
TPA (U/mg protein)	5.1 ± 0.43c	9.5 ± 0.91b	4.9 ± 0.43c	15.5 ± 1.46a	4.3 ± 0.30c	7.9 ± 0.78b	5.8 ± 1.02c	13.3 ± 1.02a
90 kDa (%)	21.3 ± 1.15a	21.2 ± 1.56ab	23.8 ± 1.69a	15.7 ± 1.84bc	32.7 ± 7.98a	13.7 ± 1.32c	15.4 ± 1.64c	16.1 ± 0.67c
60 kDa (%)	41.4 ± 4.76b	28.8 ± 2.97bc *	59.7 ± 5.41a	33.1 ± 6.77 bcd	32.2 ± 3.17bc	21.5 ± 1.57d	36.4 ± 12.22bcd	26.8 ± 2.37c
55 kDa (%)	13.6 ± 0.76bc *	18.1 ± 0.94a	14.7 ± 3.41abcd *	16.9 ± 1.69a	18.7 ± 3.87ab	11.8 ± 0.67c	22.1 ± 5.74a	8.5 ± 0.35d
50 kDa (%)	12.0 ± 2.13c *	22.2 ± 1.78b *	1.8 ± 1.31e	20.8 ± 3.87b *	4.5 ± 1.56d	28.2 ± 0.68a *	10.8 ± 2.60c	14.1 ± 0.81c
30 kDa (%)	5.1 ± 1.72bc *	4.8 ± 0.84c	0.0 ± 0.00d	5.1 ± 1.23c	4.9 ± 1.88bc	8.5 ± 0.70b *	5.0 ± 1.92bcd	11.7 ± 0.88a *
25 kDa (%)	5.2 ± 2.17bcd	4.3 ± 0.85c	0.0 ± 0.00d	6.6 ± 1.65bc	3.5 ± 1.56cd	10.2 ± 1.07ab	7.1 ±2.66abcd *	12.2 ± 1.34a
21 kDa (%)	1.0 ± 0.83c	0.2 ± 0.13c	0.0 ± 0.00c	1.6 ± 1.13bc	2.8 ± 1.45abc	4.4 ± 1.29ab	3.2 ± 1.63abc	7.0 ± 1.94a
17 kDa (%)	0.4 ± 0.44	0.5 ± 0.35	0.0 ± 0.00	0.2 ± 0.18	0.6 ± 0.46	1.0 ± 0.42	0.0 ± 0.00	1.8 ± 0.84
15 kDa (%)	0.0 ± 0.00	0.0 ± 0.00	0.0 ± 0.00	0.0 ± 0.00	0.3 ± 0.27	0.7 ± 0.37	0.0 ± 0.00	1.7 ± 0.79
Trypsin-like (%)	76.3 ± 5.87bc	68.1 ± 3.41bc	98.2 ± 1.31a	65.7 ± 7.02cd	83.6 ± 6.61ab	47.0 ± 2.95e	74.0 ±7.98bc	51.5 ± 2.89de
Chymotrypsin-like (%)	23.7 ± 5.87cd *	31.9 ± 3.41cd	1.8 ± 1.31e	34.3 ± 7.02bc	16.4 ± 6.61de	53.0 ± 2.95a *	26.0 ± 7.98cd	48.5 ± 2.89ab
**(B) Proximal Intestine**								
TPA (U/mg protein)	4.8 ± 0.6p	29.9 ± 5.5n *	10.5 ± 1.10 *	45.5 ± 4.6m *	5.9 ± 0.6p *	34.6 ± 5.6mn *	5.3 ± 0.7p	33.1 ± 4.0mn *
90 kDa (%)	17.8 ± 3.13	16.1 ± 2.61	20.1 ± 9.22	22.1 ± 1.41 *	20.2 ± 1.18	19.4 ± 0.6 *	20.0 ± 3.8	19.5 ± 0.9 *
60 kDa (%)	82.22 ± 3.13m *	24.2 ± 7.41opq	79.9 ± 9.22m	32.9 ± 5.19no	42.5 ± 6.5n	23.8 ± 0.75op	39.4 ± 7.1no	21.8 ± 0.41q
55 kDa (%)	0.0 ± 0.00p	16.3 ± 2.66mn	0.0 ± 0.00p	16.5 ± 2.26mn	11.5 ± 1.82n	17.4 ± 0.47m *	24.7 ± 6.0m	7.8 ± 0.68o
50 kDa (%)	0.0 ± 0.00o	12.4 ± 2.00mn	0.0 ± 0.00o	10.2 ± 2.55n	11.7 ± 1.74n *	13.1 ± 0.42n	9.7 ± 3.7mno	17.7 ± 1.61m *
30 kDa (%)	0.0 ± 0.00o	10.0 ± 1.72m *	0.0 ± 0.00o	5.8 ± 1.66n	4.4 ± 1.22n	6.7 ± 0.44n	6.3 ± 3.3mno	6.4 ± 0.39n
25 kDa (%)	0.0 ± 0.00o	16.4 ± 2.91m *	0.0 ± 0.00o	8.0 ± 2.12n	5.4 ± 1.79n	14.5 ± 0.44m *	0.0 ± 0.00o	15.5 ± 0.62m
21 kDa (%)	0.0 ± 0.00p	2.6 ± 1.15no *	0.0 ± 0.00p	1.0 ± 0.46op	3.1 ± 1.32mnop	4.8 ± 0.42n	0.0 ± 0.00p	7.0 ± 0.38m
17 kDa (%)	0.0 ± 0.00n	1.1 ± 0.75mn	0.0 ± 0.00n	1.0 ± 0.45m	0.6 ± 0.37mn	0.0 ± 0.00n	0.0 ± 0.00n	1.6 ± 0.55m
15 kDa (%)	0.0 ± 0.00n	0.9 ± 0.54mn	0.0 ± 0.00n	2.5 ± 0.81m *	0.6 ± 0.37mn	0.1 ± 0.08n	0.0 ± 0.00n	2.8 ± 1.00m
Trypsin-like (%)	100.0 ± 0.00m *	56.7 ± 7.75op	100.0 ± 0.00m	71.5 ± 7.37no	74.2 ± 5.79no	60.7 ±1.08o *	84.0 ±6.9mn	49.1 ± 1.00p
Chymotrypsin-like (%)	0.0 ± 0.00p	43.3 ± 7.75mn	0.0 ± 0.00p	28.5 ± 7.37no	25.8 ± 5.79no	39.3 ± 1.08n	16.0 ±6.87op	50.9 ± 1.00m

#### 3.1.3. HT R- Versus HF R- and HT ST-Fed Gilthead Sea Bream

If we focus first on anticipatory digestive enzyme activity, in the pyloric caeca, HT R fish showed the highest lipase activity (Figure 2A), and α-amylase activity was similar to that of HT ST gilthead sea bream and higher than that of HF R fish (*p*-value = 0.034) (Figure 2B). In addition, comparable TPA activity was found among the three groups (Figure 2E). However, the trypsin/chymotrypsin ratio was lowest in the HT R group due to changes in activities of the 90, 60, and 50 kDa bands (*p*-value < 0.05) (Figure 3 and Table 3).

In contrast, in the proximal intestine, lipase activity was similar among the HT ST, HF R, and HT R groups (Figure 2D). The HT R fish also showed intermediate α-amylase activity and lower TPA activity than that in HT ST animals (*p*-value ˂ 0.001) (Figure 2C,E). The trypsin/chymotrypsin ratio was intermediate in HT R animals compared to HT ST and HF R, with trypsin-like (60 and 55 kDa, *p*-value < 0.05) and chymotrypsin-like (50, 30, and 25 kDa) activities involved in these changes (Figure 3 and Table 3).

Focusing on postprandial digestive activity in the pyloric caeca, HT R animals showed intermediate lipase activity compared to HT ST- and HF R-fed fish (*p*-value = 0.018 and 0.033, respectively) (Figure 2A) and an α-amylase activity similar to HF R gilthead sea bream and lower than that of HT ST animals (*p*-value ˂ 0.001) (Figure 2B). Moreover, TPA activity was similar to the HT ST group and higher than the HF R group (*p*-value ˂ 0.001) (Figure 2C), but with a trypsin/chymotrypsin ratio similar to HF R fish. Activities of proteases 60, 55, 50, 30, 25, and 21 kDa were modified among the three groups (Figure 3 and Table 3).

In contrast, in the proximal intestine, lipase, α-amylase and TPA activities were similar among HT ST, HF R, and HT R groups (Figure 2D–F), but HT R fish had the lowest trypsin/chymotrypsin ratio, being less than 1, with trypsin-like (60 and 55 kDa, *p*-value < 0.05) and chymotrypsin-like (50, 25, 21, 17, and 15 kDa, *p*-value ˂ 0.05) activities involved in these changes (Figure 3 and Table 3).

Both gilthead sea bream fed under dietary restriction tended to increase their RIL compared to fish fed standard ration (0.63 ± 0.04 for HF R and 0.68 ± 0.04 for HT R versus 0.59 ± 0.04 for HT ST), possibly as a compensatory mechanism to improve digestion but without achieving the same growth as fish fed the standard ration.

### 3.2. Intestinal Microbiota Assessment

**Figure 4:**
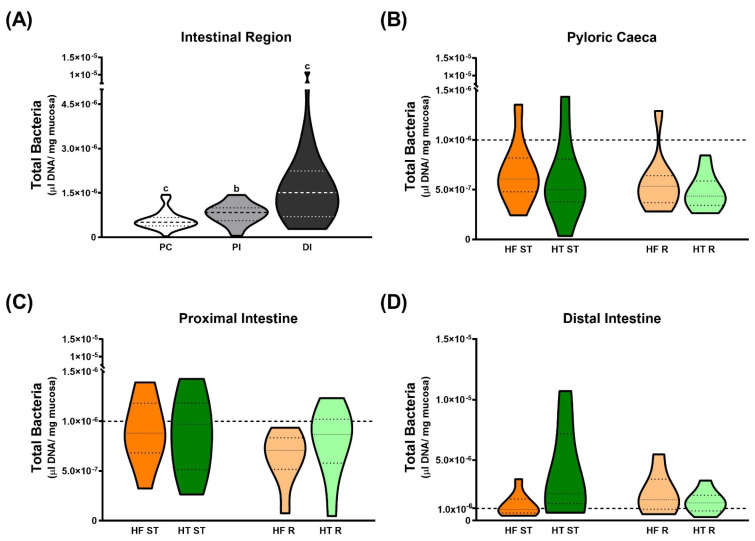
Total bacteria (µL DNA/ mg mucosa) (**A**) by intestinal region, (**B**) in pyloric caeca, (**C**) in proximal intestine, and (**D**) in distal intestine, determined by qPCR in gilthead sea bream fed the experimental diets for 8 weeks. To facilitate comparison between the graphs, the dotted line was established at the concentration of 1.0 × 10^−6^ bacteria in all graphs. (**A**) Comparisons were performed using Kruskal–Wallis and Dunnett’s tests. Data are shown as mean ± S.E.M (n = 27). Significant differences between intestinal regions in (**A**) are shown by different letters (*p*-value < 0.05). In (**B**–**D**), comparisons were performed using two-way ANOVA and Sidak’s tests. Data are shown as mean ± S.E.M (n = 9).

A significant increase was detected along the intestinal tract when comparing total bacteria among intestinal regions (*p*-value ˂ 0.001) (Figure 4A). However, no changes in total bacteria concentration were found in response to either dietary treatment or ration in any of the studied regions (Figure 4B–D).

**Figure 5:**
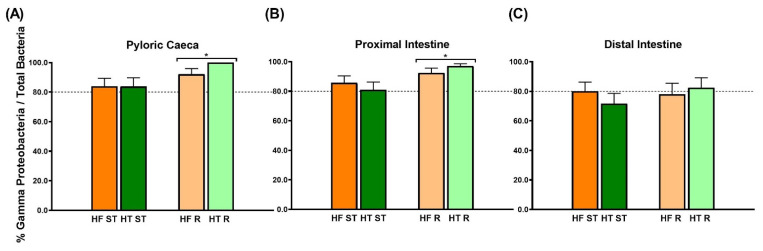
γ-proteobacteria population related to total bacteria concentration in (**A**) pyloric caeca, (**B**) proximal intestine and (**C**) distal intestine determined by qPCR in gilthead sea bream fed the experimental diets for 8 weeks. The dotted line was established at 80% in all graphs in accordance with the mean values for this phylum in marine fish species [50,51]. Data are shown as mean ± S.E.M (n = 9). Comparisons were performed using two-way ANOVA and Sidak’s tests. Significant differences between standard ration-fed fish and restricted-fed fish are shown by an asterisk (*p* < 0.05).

γ-Proteobacteria, a bacterial class belonging to the Proteobacteria phylum, ranged from 71.7 to 99.5% across all experimental groups and intestinal regions (Figure 5). The results showed that dietary restriction enhanced γ-Proteobacteria population growth in the pyloric and proximal intestinal regions (*p*-value = 0.019 and *p*-value = 0.027, respectively) (Figure 5A,B), whereas no ration effect was detected in the distal intestine (Figure 5C).

Actinobacteria and Firmicutes phyla were also determined by qPCR, but their concentrations were too low to provide reliable results. In addition, Enterobacteria, a taxonomical subgroup of γ-Proteobacteria, were also assessed; however, as with the previous phyla, the proportions obtained were very low.

## 4. Discussion

Plant feed additives for livestock nutrition have gained increasing attention since they are rich in bioactive compounds, like polyphenols, conferring healthy properties [70,71]. In the present study, digestive processes in gilthead sea bream fed a high-fat diet at standard or restricted ration, in which a hydroxytyrosol-rich olive oil extract was included, were evaluated.

Hydroxytyrosol, oleuropein, and tyrosol are the main phenolic compounds present in olive oil [72]. While their health-promoting properties are well known in mammals, their effects as functional feed additives in fish remain largely unknown. After feeding, these compounds were mostly found in the intestinal lumen and showed natural anti-inflammatory and high antioxidant activities [28,29]. In the present study, their antioxidant properties were also assessed in the two intestinal segments studied showing a reduction of malondialdehyde levels by 38.9% in pyloric caeca and 15.9% in proximal intestine (unpublished data), according to those well-known antioxidant properties of polyphenols in mammals, poultry, and fish [44,48,73,74,75,76].

To our knowledge, limited information regarding both anticipatory and digestive enzyme activities in fish fed diets containing polyphenols is available. The present results showed a significant increase in digestive lipase activity in the pyloric caeca region in gilthead sea bream fed HT diets, both at standard and restricted rations (to a lower degree), whereas in the proximal intestine, this increase was only found at 24 h post-feeding. Accordingly, an increase in lipase activity was also found in hybrid juvenile sturgeon (*Acipenser Baerii ♀ × A. Schrenckii ♂*) fed with diets containing tea polyphenols [48], and in common carp (*Cyprinus carpio*) fed with diets containing olive leaf extract [39,77], whereas no changes were found in rainbow trout fed with diets containing olive mill vegetation water [78]. An upregulation of this enzyme activity was found in fish fed with low-lipid diets, acting as a compensatory mechanism to improve lipid digestion and absorption [79,80]; but this could also be found in fish fed with diets with high lipid, low carbohydrate, and moderate-low protein content [81]. Nevertheless, in the present study, dietary protein content met gilthead sea bream requirements properly, and those changes were more important in the pyloric caeca than in the proximal intestine, as pancreatic enzyme release takes place in this region of the intestinal tract. Recent studies in gilthead sea bream have shown better growth performance and reduced perivisceral, hepatic, and intestinal lipid deposits by dietary inclusion of bile salts related to a higher dietary bile-salt activated lipase [82], improving lipid digestion [83]. Similar results were also found in tongue sole (*Cynoglossus semiliaevis*) [84], largemouth bass (*Micropterus salmoides*) [85], and yellow croaker (*Larimichthys crocea*) [86]. This high lipase activity in gilthead sea bream fed HT diets could also be related to a higher bile acid production, as previously reported in dogs by Yago et al. [87] after 8 months of feeding with a diet containing olive oil. Balbuena-Pecino et al. [60] also demonstrated body adiposity modulation by hydroxytyrosol dietary inclusion in gilthead sea bream, suggesting a link between this polyphenol inclusion and bile acid production. In agreement with these promising results, dietary HT induced a high musculoskeletal growth potential, although no significant differences in growth were observed, possibly due to the HT dose or study duration [61]. In this sense, growth improvement was also not found in the marbled spinefoot rabbitfish (*Siganus rivulatus*) [88] nor in Atlantic salmon [89] fed bile salt-supplemented diets.

Moreover, polyphenols present in olive oil, like HT and other bioactive compounds, can bind and precipitate enzymes through non-covalent interactions, as well as proteins and polysaccharides, forming complexes that delay food digestion and even reduce their digestibility, as has been described for tea polyphenols [90,91,92,93]. In this sense, we hypothesize that the upregulated activity of α-amylase found in gilthead sea bream fed HT diets may be a compensatory mechanism for the low glucose availability, on one hand due to the low starch dietary content and on the other hand to polyphenol-induced inhibition by both starch binding sites and α-amylase activity, as *in vitro* studies have demonstrated [90,91,93,94,95,96]. Similar results regarding α-amylase activity were found in hybrid sturgeon fed tea polyphenols in a dose-dependent manner [48] and in common carp [77] fed diets with olive leaf extract, pointing to a higher activity at low polyphenol concentrations, while non-competitive inhibition at high concentrations [90,92]. A reduction in intestinal transit rate has also been observed in fish fed high-lipid diets [97], which may contribute to the regulation of glucose levels since no changes in plasma glucose levels were found in the present study between experimental groups (see Balbuena-Pecino et al. [60]), probably related to the low carbohydrate dietary content.

In mammals, olive oil dietary inclusion is a potent stimulus of cholecystokinin (CKK) release [98,99]. In the present study, TPA significantly rose during digestion in gilthead sea bream fed HT diets, regardless of the given ration, suggesting that in fish, olive extract inclusion could act as a CCK secretagogue, as observed in mammals. This idea agrees with the higher α-amylase and lipase activities previously described. Similar results were found in juvenile hybrid sturgeon fed with diets containing tea polyphenols [48], in common carp fed with low doses of olive leaf extract [77], and in Mozambique tilapia fed with a diet supplemented with curcumin [100]. Instead, in rainbow trout, only pepsin activity was modified by olive oil dietary inclusion [78], pointing to clear species-specific effects. Furthermore, Yago et al. [87] demonstrated in dogs that olive oil not only stimulates CKK but also contributes to YY peptide (PYY) and pancreatic polypeptide (PP) release in the distal intestine, inhibiting both pancreatic secretions and reducing gastric emptying and thereby increasing digestion time compared to animals fed diets containing sunflower oil. In the present study, gilthead sea bream under dietary restriction tended to increase RIL versus those fed standard ration, being higher for those fed with HT R, suggesting that those fish presented a lower intestinal transit than HF R.

In the present study, zymograms revealed the activity of nine proteases in gilthead sea bream for the first time. Six of them, three with trypsin-like (90, 60, and 55 kDa) and three with chymotrypsin-like activity (50, 30, and 25 kDa), previously characterized and commonly found in this species, showing a different profile depending on diet, ration, and post-feeding time [62,79,81,97]. Moreover, chymotrypsin-like activities of 21, 17, and 15 kDa were detected in this species for the first time, suggesting that an HFD compromised protein digestion since this upregulation in protease activity has been previously described as a compensatory mechanism to improve digestion [80,97,101,102]. Accordingly, low molecular weight protease activities were also detected in the proximal intestine of sea bass (*Dicentrarchus labrax*) fed high-lipid (22% lipids) or high-carbohydrate (more than 29% starch) diets 24 h post-feeding [81]. In pyloric caeca, dietary restriction is the main factor affecting individual protease activities after feeding, showing a significant diminution in 55 kDa trypsin-like activity percentage compensated by an increase in 30, 25, and 21 kDa chymotrypsin-like activities, leading to an apparent effect of HT dietary inclusion on TPA. Despite this, the changes in chymotrypsin-like release did not modify the trypsin/chymotrypsin ratio, probably due to the dietary effect on the 50 kDa protease release, a chymotrypsin-like protease with higher activities during digestion in gilthead sea bream fed with HF diets. Individual protease activities detected by zymography in the proximal region showed a completely different regulation pattern than those found in pyloric caeca [103,104]. Thus, in the proximal intestine, both trypsin-like and chymotrypsin-like anticipatory activities were affected by restriction but with a similar TPA activity, pointing to a compensatory mechanism in both trypsin-like and chymotrypsin-like release. The downregulation of 60 and 55 kDa trypsin-like activities and the upregulation of 50 and 30 kDa chymotrypsin-like activities at 24 h post-feeding confer fish an adaptative advantage to improve digestion, as previously reported in rats [105]. Moreover, during digestion, 21 kDa activity was also enhanced by dietary restriction. Unlike in pyloric caeca, the proximal intestine showed a clear protease modulation related to diet, mainly during digestion, where lower 55 and 25 kDa activities and higher 15 kDa activity were detected in gilthead sea bream fed HT diets compared to HF-fed fish. Considering that trypsin and chymotrypsin cut proteins through different sites, releasing different amino acids and peptides [106], changes in their proportions could influence digestion due to variations in amino acid availability [97].

Concerning the composition of the gut microbiota, it has been widely observed that there exists a gradual increase in the bacterial concentration from the stomach toward the posterior intestine in fish [107], which agrees with the results found in the present study (Figure 4A). Moreover, the percentage of γ-Proteobacteria in this study was approximately 80% in all regions, verifying the prevalence of the Proteobacteria phylum as previously reported [50,51]. A comparable prevalence in the gut microbiota of marine fishes was reported by Sullam et al. [108], with the vast majority of the analyzed Proteobacteria classified as γ-Proteobacteria in the case of carnivorous marine fishes. Regarding the dietary factors that can modulate gut microbiota composition, Proteobacteria was maintained as the dominant phylum during fasting in the leopard coral grouper [109], while the hybrid grouper presented a significant overall reduction in intestinal microbial abundance and diversity under starvation [110]. Moreover, Tran et al. [111] demonstrated a different microbiota modulation comparing feeding and starvation in grass carp. These last results are in line with those found in the present study in gilthead sea bream exposed to a dietary restriction. Despite this, the differences detected in pyloric caeca and proximal intestine disappeared in the distal region. Similar results were obtained by Ruiz et al. [112] in the intestine of gilthead sea bream fed diets supplemented with bile acid salts. Regarding the effects of HT dietary inclusion, Liu et al. [113] did not find significant changes in Proteobacteria in mice, suggesting that HT may prevent obesity through other mechanisms of gut microbiota modulation, possibly through minor subgroups. More studies using high-resolution techniques, like Next-Generation Sequencing, on the effect of dietary supplementation with HT on fish gut microbiota are needed since their relationship with host energy and lipid metabolism has not been elucidated yet.

## 5. Conclusions

This study provides the first validation of hydroxytyrosol’s digestive function in fish fed with high-fat diets. The inclusion of an olive oil extract containing this polyphenol in the diet of gilthead sea bream upregulated digestive enzyme activities, specifically trypsin-like activities, in both the pyloric caeca and the proximal intestine. Instead, restricted feeding mainly modulated digestion in the pyloric caeca, increasing chymotrypsin-like activities, tending to increase the RIL, and enhancing the growth of γ-proteobacteria in the pyloric caeca and proximal intestine. Therefore, dietary hydroxytyrosol inclusion at a standard ration may improve digestion in gilthead sea bream fed high-fat diets in healthier conditions than without this additive. However, further research is needed to determine optimal supplementation levels and to assess the long-term safety and efficacy of hydroxytyrosol in gilthead sea bream and other aquaculture species commonly fed high-fat diets.

## Figures and Tables

**Table 1:** Dietary composition of the high-fat diet (HF) and the high-fat diet supplemented with hydroxytyrosol (HT). The hydroxytyrosol was included in the formulation at a dose of 1.66 g HIDROX^®^/kg feed (0.52 g hydroxytyrosol/kg feed). This extract is obtained from olive juice (Oliphenol LLC., Hayward, CA, USA) and contains >12% simple and total polyphenols: 3.136% hydroxytyrosol.

	HF	HT
**Ingredients (%)**		
Corn gluten	3.80	3.80
Wheat gluten	20.00	20.00
Fava beans	8.00	8.00
Soya concentrate	25.00	25.00
Fish meal	15.00	15.00
Fish oil	9.98	9.98
Rapeseed oil	10.14	10.14
Yttrium premix	0.10	0.10
Phosphate	1.04	1.04
Vitamin mineral premix	0.44	0.44
Wheat	6.50	4.85
HIDROX^®^	0	1.66
**Composition (%)**		
Dry matter	93.0	93.0
Moisture	7.0	7.0
Crude protein	46.8	46.7
Crude fat	24.0	24.2
Ash	5.4	5.6
Crude fiber	1.9	1.8
Starch	8.8	7.8

**Table 2:** Targeted primer sequences (AN: annealing temperature; F: forward; R: reverse) [66,67,68].

Total Bacteria (AN: 65 °C)	F: ACT CCT ACG GGA GGC AGC AGT R: ATT ACC GCG GCT GCT GGC
γ-Proteobacteria (AN: 54 °C)	F: GCT CGT GTT GTG AAA TGT TGG R: CGT AAG GGC CAT GAT GAC TTG
Actinobacteria (AN: 54 °C)	F: TAC GGC CGC AAG GCT A R: TCR TCC CCA CCT TCC TCC G
Firmicutes (AN: 52 °C)	F: CTG ATG GAG CAA CGC CGC GT R: ACA CYT AGY ACT CAT CGT TT
Enterobacteriaceae (AN: 60 °C)	R: ATG GCT GTC GTC AGC TCG T F: CCT ACT TCT TTT GCA ACC CAC T

## Data Availability

The raw data supporting the conclusions of this article will be made available by the authors on request.
